# The Fibrosis-Targeted Collagen/Integrins Gene Profile Predicts Risk of Metastasis in Pulmonary Neuroendocrine Neoplasms

**DOI:** 10.3389/fonc.2021.706141

**Published:** 2021-08-11

**Authors:** Tabatha Gutierrez Prieto, Juliana Machado-Rugolo, Camila Machado Baldavira, Ana Paula Pereira Velosa, Walcy Rosolia Teodoro, Alexandre Muxfeldt Ab´ Saber, Vera Luiza Capelozzi

**Affiliations:** ^1^Laboratory of Genomics and Histomorphometry, Department of Pathology, University of São Paulo Medical School (USP), São Paulo, Brazil; ^2^Health Technology Assessment Center (NATS), Clinical Hospital (HCFMB), Medical School of São Paulo State University (UNESP), Botucatu, Brazil; ^3^Rheumatology Division of the Clinical Hospital, University of São Paulo Medical School (USP), Sao Paulo, Brazil

**Keywords:** collagens, integrins, extracellular matrix, metastasis, pulmonary neuroendocrine neoplasms, surgical resection

## Abstract

Recently, collagen/integrin genes have shown promise as predictors of metastasis mainly in non-small cell lung cancer and breast cancer. However, it is unknown if these gene expression profiling differ in metastatic potential of pulmonary neuroendocrine neoplasms (PNENs). In this study, we sought to identify differentially expressed collagen/integrin genes in PNENs in order to understand the molecular mechanisms underlying the development of stroma-associated fibrosis for invasion and metastasis. We compared collagen/integrin gene expression profiling between PNE tumors (PNETs) and PNE carcinomas (PNECs) using a two-stage design. First, we used PCR Array System for 84 ECM-related genes, and among them, we found *COL1A2*, *COL3A1*, *COL5A2*, *ITGA5*, *ITGAV*, and *ITGB1* functionally involved in the formation of the stroma-associated fibrosis among PNENs histological subtypes. Second, we examined the clinical association between the six collagen/integrin genes in tumor tissues from 24 patients with surgically excised PNENs. However, the pathological exam of their resected tissues demonstrated that 10 developed lymph node metastasis and 7 distant metastasis. We demonstrated and validated up regulation of the six fibrogenic genes in PNECs and down regulation in PNETs that were significantly associated with metastasis-free and overall survival (P<0.05). Our study implicates up regulation of fibrogenic genes as a critical molecular event leading to lymph node and distant metastasis in PNENs.

## Introduction

Neuroendocrine neoplasms (NENs) are classified into differentiated neuroendocrine tumors (NETs), also known as carcinoid tumors (typical carcinoid and atypical carcinoid), and poorly differentiated neuroendocrine carcinomas (NECs), including large cell neuroendocrine carcinoma (LCNEC) and small cell carcinoma (SCLC) (1). Patients with pulmonary neuroendocrine tumors (PNETs) have tumors sufficiently localized to be considered treatable by surgical resection, and among those whose tumors are successfully resected, approximately 90-98% of patients with typical carcinoid, and 50-60% of atypical carcinoid, survive 5 years (2, 3). In contrast, only 20-30% of the patients with large cell neuroendocrine carcinoma survive 5 years after surgical resection and adjuvant chemotherapy (4), and only 10% of the patients with small cell lung carcinoma survive 5 years after Cisplatin + Carboplatin + Etoposide (5). Clearly, some PNETs and PNECs have developed occult dissemination beyond the lung even when they appear to have been completely removed or responsive to adjuvant chemotherapy. Since different PNENs are composed of different mutated neuroectodermic cells, their malignant potential and prognosis may vary greatly. Although some of these differences are known to physicians, not only is it still often difficult to predict which tumors will invade, metastasize, and shorten the patient’s life, but effective adjuvant treatments still depend on identifying these tumors shortly after biopsy or surgery as well.

Due to tissue accessibility, genome-wide examination of biomarkers associated with metastatic progression and cancer specific death has primarily been based on observations made in the primary tumor behavior (6–8) and not their effects on the stroma-associated fibrosis more lethal, and more therapeutically relevant for metastatic lesion. In addition, genome-wide studies that have preliminarily explored in metastatic tumors have done so using small sample sizes (9–13). Thus, the molecular mechanisms that lead to PNENs metastasis remain largely unknown and require further study. The identification of fibrogenic genes in primary tumors and their effects on tumor microenvironment (TME) as new biomarkers and therapeutic targets for PNENs is promising.

To address these gaps in the knowledge, we identified fibrotic genes that support PNENs metastasis in localized surgically resected primary tumor. Overall, we performed an analysis of gene expression data generated using mRNA in two approaches where we first utilized gene expression microarray technology to identify candidate genes that are associated with PNENs metastasis, and subsequently, validated the candidate genes in a similar cohort of patients with PNENs tumors using *in silico* analysis.

## Methods

### Patient Selection

#### Discovery Cohort

We identified 24 patients at A. C. Camargo Cancer Center, in São Paulo, Brazil and Hospital do Amor, in Barretos, Brazil, who were surgically resected with PNENs [10 carcinoid tumors (5 TC and 5 AC)], 4 LCNEC, 10 SCLC, and had fresh-frozen tissue available from their primary tumor.

Two pathologists (T.G.P. and V.L.C) carried out a blinded comprehensive review of all tumors to confirm histological subtype (14), the mitotic count, the presence of an organoid pattern (rosettes, pseudo rosettes, palisading, spindle cells) and necrosis. The neoplastic area was delimited during the frozen section procedure to ensure the exclusion of non-neoplastic tissue. Patient’s demographics and clinicopathological characteristics were obtained from medical records and included age, sex, smoking history, tumor size, tumor stage (according to the International Association for the Study of Lung Cancer classification system, 8th edition), and follow-up information (14).

The internal ethics committees of all the participating institutions approved this study’s protocol (process number 1.077.100) with a waiver for informed consent by their review boards.

#### Validation Cohort

To validate our data we performed *in silico* analysis using public database. Data included 19 normal lung tissues, 8 LCNEC tissues, 15 SCLC tissues, and 12 primary typical carcinoids. The GSE1037 gene expression profile was obtained from the National Center for Biotechnology Information Gene Expression Omnibus^1^ (GEO) (15, 16) based on the GPL962 platform. The mRNA expression raw data were analyzed by GEO2R online tools. In order to compare gene expression, we created two heatmaps using the Heatmapper platform^2^ to investigate the gene expression of 5 different genes, namely *COL1A2* (α2 chain *COL I*), *COL3A1 *(α1 chain *COL III*), *COL5A2* (α2 chain *COL V*), *ITGA5* (alpha 5-integrin) and *ITGB1* (beta 1-integrin), in each PNEN histological type, comparing the profile seen in GSE1037 to that of our cohort. Then, we used the average distance and the Euclidean distance between elements to perform an unsupervised hierarchical grouping.

### Gene Expression Profile Data

The neoplastic area was micro dissected during the frozen section procedure to ensure the inclusion of neoplastic tissue and distant non-neoplastic tissue as control. Total mRNA was extracted from fresh-frozen tumor and normal tissues using the QIAsymphony miRNA CT 400 kit (Qiagen, CA, USA) according to the manufacturer’s instructions. RNA integrity and quality were determined using the Bioanalyzer 2100 (Agilent Technologies). Complementary DNA was synthesized using the c-DNA – RT² First Strand Kit (Qiagen Sample & Assay Technologies) according to the manufacturer’s protocol. The difference of expression in EMT genes was evaluated by the real-time PCR method. Quantitative reverse transcription-polymerase chain reaction (qRT-PCR) was performed using the RT² Profiler PCR Array System (PAHS-090Z; Qiagen, Dusseldorf, Germany) kit for the human epithelial-to-mesenchymal transition (EMT) pathway with 84 target genes. The array includes a total of 84 EMT genes, 5 housekeeping genes (*ACTB*, *B2M*, *GAPDH*, *HPRT1*, *RPLP0*), 1 genomic DNA control (GDC) to assess contamination, 3 reverse transcriptase controls (RTC) that certify the efficiency of the reverse transcription step, and 3 positive PCR controls (PPC) consisting of an artificial DNA sequence certifying the test accuracy. Each 96-well plate includes SYBR^®^ Green-optimized primer assays for a thoroughly researched panel of 84 EMT genes, that also are included the collagen and integrin genes. Furthermore, the high-quality primer design and RT^2^ SYBR^®^ Green qPCR Mastermix formulation enable the PCR array to amplify 96 gene-specific products simultaneously under uniform cycling conditions. The samples were amplified using Applied Biosystems Step One Plus (Applied Biosystems, California, USA). The cycling conditions were as follows: 95°C for 10 minutes, 40 cycles at 95°C for 15 seconds, 60°C for 1 minute, followed by the dissociation period. The data were then analyzed in the StepOne software (v. 2.0, Applied Biosystems) using the Δ threshold cycle (Ct) method (2^−ΔΔCt^) (17). All data were normalized by the housekeeping genes, and normal lung tissue specimens were used as case control. Using the EMT expression analysis, we created a heatmap of EMT gene expression across PNENs histological subtypes, which showed different levels of expression between 6 EMT genes (FC≥ 2.0). Among them, we found *COL1A2*, *COL3A1*, *COL5A2*, *ITGA5, ITGAV* and *ITGB1* that were differentially expressed among PNENs histological subtypes.

### Functional Enrichment Analysis of Collagen and Integrin Genes

To further elucidate the function and signaling pathways involved in the enrichment of the collagen and integrin genes, we inputted the selected genes plus *ITGAV*, totaling 6 genes, into Metascape (18) to perform Gene Ontology (GO) function, KEGG, and REACTOME pathway analyses. The GO analysis was composed of 3 categories, namely, biological processes (BPs), cellular components (CCs), and molecular functions (MFs). Results that met the threshold value with P<0.05 were regarded as significant.

### PPI Network Construction and Module Analysis

To reveal the functional interactions among the proteins encoded by these genes, the selected genes were uploaded into STRING tools to map their PPI network (18). Results that presented a combined interaction score of P>0.9 were considered to be significant.

### Data Management and Statistical Analysis

Data were collected and managed using REDCap electronic data capture tools hosted at A. C. Camargo Cancer Center, in São Paulo, Brazil. Considering the non-normal distribution of our data, all statistical tests employed in this study to examine the difference between categories and groups were non-parametric tests. The chi-square test or Fisher’s exact test, the non-parametric Kendall tau-b correlation coefficient and the Spearman’s rank correlation coefficient were used to examine differences in categorical variables, whereas the Kruskal-Wallis test was used to detect differences in continuous variables between groups of patients. However, to analyze the demographic and clinicopathological characteristics of the patients, the Person’s Chi-Square test was used for these categorical variables. Qualitative data were described using relative frequencies. Overall survival (OS) was defined as the interval from the date of biopsy or surgical resection to death and OS curves were estimated using the Kaplan–Meier method. The Cox proportional hazards model was then used to analyze the association between OS rate and other covariances, and only parameters that presented P ≤ 0.02 in a univariate analysis were considered for multivariate analyses. We used the Statistical Package of Social Science (SPSS) version 18 for all statistical analyses. All tests with P<0.05 were deemed statistically significant and a Bonferroni correction was used when necessary.

## Results

### Discovery Cohort: Differential Gene Expression Profiling of Primary PNENs

**Table 1** summarizes the clinical characteristics of patients, stratified by histological types. LCNEC and AC tended to be more frequent in female than male patients (3, 75.0% and 4, 80.0%). As for median age, a similar distribution was found across all histologic types. As expected, a history of tobacco smoking was more associated with SCLC when compared to LCNEC and carcinoid tumors, with statistical significance. Pathological stage showed a difference among the PNETs and PNECs histotypes. While all patients with carcinoid tumors were in an early stage of the disease, those with neuroendocrine carcinomas (SCLC and LCNEC) were in an advanced stage (P<0.01). Before surgical resection, patients with SCLC (3, 30.0%) and LCNEC (1, 25.0%) received prophylactic radiotherapy. Pathological examination of the surgically resected tumor detected lymph node metastasis in 6 SCLC [N2, 2 (33.3%); N3, 4 (66.7%)], in 3 LCNEC [N1, N2 and N3, with 1 (25.0%) each one], and 1TC [N1, 1(33.3%)]. During follow up, patients with SCLC (6, 60.0%) and LCNEC (1, 33.3%) developed distant metastasis. Systemic chemotherapy was indicated for patients with SCLC (8, 80.0%), LCNEC (3, 75.0%), AC (2, 50.0%) and TC (2, 66.7%). The median follow-up of the patients was 30 (1–168). Regarding the status, 14 patients died (10, SCLC; 3, LCNEC and 1, AC) and 10 (1, LCNEC; 4, AC and 5, TC) are still alive.

**Table 1:** Demographic and clinicopathologic characteristics of patients diagnosed with PNENs by Person’s Chi-Square test (P<0.05).

Characteristics	Histological types				*P*-value
	TC (N=5)	AC (N=5)	LCNEC (N=4)	SCLC (N=10)	
**Gender**					0.354
*Male*	3 (60.0%)	1 (20.0%)	1 (25.0%)	6 (60.0%)	
* Female*	2 (40.0%)	4 (80.0%)	3 (75.0%)	4 (40.0%)	
**Age (median, yrs)**					0.215
* <56*	3 (60.0%)	4 (80.0%)	1 (25.0%)	3 (30.0%)	
* ≥ 56*	2 (40.0%)	1 (20.0%)	3 (75.0%)	7 (70.0%)	
**Smoke Status**					0.003
* Yes*	1 (20.0%)	1 (20.0%)	3 (75.0%)	10 (100%)	
* No*	4 (80.0%)	4 (80.0%)	1 (25.0%)	0 (0.0%)	
**Prophylatic Radiotherapy**					0.472
* Yes*	0 (0.0%)	0 (0.0%)	1 (25.0%)	3 (30.0%)	
* No*	3 (100%)	4 (100%)	3 (75.0%)	7 (70.0%)	
**Stage at tumor analyses**					
**^a^T** **Stage†**					0.085
* T1*	1 (33.3%)	2 (66.7%)	0 (0.0%)	0 (0.0%)	
* T2*	1 (33.3%)	0 (0.0%)	2 (50.0%)	1 (16.7%)	
* T3*	0 (0.0%)	1 (33.3%)	0 (0.0%)	0 (0.0%)	
* T4*	1 (33.3%)	0 (0.0%)	2 (50.0%)	5 (83.3%)	
**^a^N** **Stage†**					0.088
* N0*	2 (66.7%)	3 (100%)	1 (25.0%)	0 (0.0%)	
* N1*	1 (33.3%)	0 (0.0%)	1 (25.0%)	0 (0.0%)	
* N2*	0 (0.0%)	0 (0.0%)	1 (25.0%)	2 (33.3%)	
* N3*	0 (0.0%)	0 (0.0%)	1 (25.0%)	4 (66.7%)	
**^a^M** **Stage†**					0.121
* M0*	3 (100%)	3 (100%)	2 (66.7%)	4 (40.0%)	
* M1*	0 (0.0%)	0 (0.0%)	1 (33.3%)	6 (60.0%)	
**Pathological stage**					0.000
* Early stage (I/II)*	5 (100%)	5 (100%)	0 (0.0%)	0 (0.0%)	
* Advanced stage (III/IV)*	0 (0.0%)	0 (0.0%)	4 (100%)	10 (100%)	
**Systemic Chemotherapy**					0.725
* Yes*	2 (66.7%)	2 (50.0%)	3 (75.0%)	8 (80.0%)	
* No*	1 (33.3%)	2 (50.0%)	1 (25.0%)	2 (20.0%)	
**Status**					0.001
* Alive*	5 (100%)	4 (80.0%)	1 (25.0%)	0 (0.0%)	
* Dead*	0 (0.0%)	1 (20.0%)	3 (75.0%)	10 (100%)	
***Follow-up*** *(months)*	30 (1 – 168)				–

PNENs, pulmonary neuroendocrine neoplasms; TC, typical carcinoid; AC, atypical carcinoid; LCNEC, large cell neuroendocrine carcinoma; SCLC, small cell lung carcinoma.

a Some cases lacked follow-up information: T stage (8); N stage (8); M stage (5); Chemotherapy and Radiotherapy (3).

^†^8th International Association for the Study of Lung Cancer (14).

**Figure 1:**
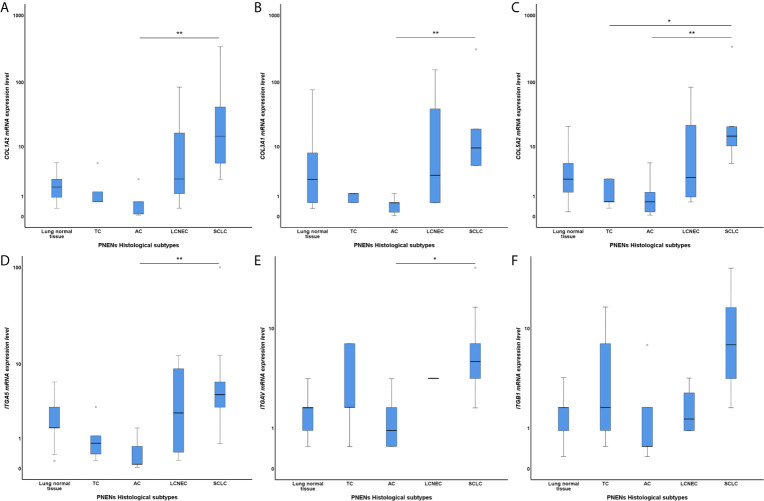
Box plots of differentially expressed *COL* and *ITG* gene expressions among PNENs histological subtypes in a log scale. The top and bottom of the box plot represents the 25th and 75th percentile range. The line across the box shows the median of gene expression and the top and bottom bars show the maximum and minimum values, outliers were showed. The association between *COL* and *ITG* gene expressions and PNENs histological types was calculated by non-parametric Kruskal-Wallis test. **(A)** Difference of *COL1A2* mRNA expression level among PNENs histological subtypes (P=0.004; Adjusted P-value [AC-SCLC]=0.007); **(B)** Difference of *COL3A1* mRNA expression level among PNENs histological subtypes (P=0.004; Adjusted P-value [AC-SCLC]=0.004); **(C)** Difference of *COL5A2* mRNA expression level among PNENs histological subtypes (P=0.003; Adjusted P-value [AC-SCLC]=0.010; Adjusted P-value [TC-SCLC]=0.019); **(D)** Difference of *ITGA5* mRNA expression level among PNENs histological subtypes (P=0.009; Adjusted P-value [AC-SCLC]=0.008); **(E)** Difference of *ITGAV* mRNA expression level among PNENs histological subtypes (P=0.028; Adjusted P-value [AC-SCLC]=0.018); **(F)** Difference of *ITGB1* mRNA expression level among PNENs histological subtypes (P=0.057). The horizontal bars represents the statistical difference between groups, the pairwise comparison was adjusted by Bonferroni’s correction for multiple tests (Adjusted P-value). (*) was used when P > 0.05, and (**) was used when P ≥ 0.01. SCLC, small cell lung carcinoma; LCNEC, large cell neuroendocrine carcinoma; AC, atypical carcinoid; TC, typical carcinoid.

**Table 2** summarizes the distribution of the six genes studied in our cohort among histological types. Five of them – *COL1A2*, *COL3A1*, *COL5A2*, *ITGA5*, and *ITGAV* – showed a similar pattern of gene expression. Their expression increased as we moved from normal lung tissue to LCNEC and SCLC, where they were overexpressed, and decreased from normal lung tissue to carcinoid tumors, where they were downregulated (P<0.05). However, *ITGB1* differed from the rest of the set and was overexpressed only in SCLC patients. In LCNEC and AC, its expression was lower than that of normal lung tissue and, in TC, expression was almost equal to that of normal lung tissue. The **Figure 1** shows the box plots of *COL* and *ITG* gene expressions among PNENs histological subtypes.

**Table 2:** Association between gene expression of *COL* and *ITG* and PNENs histological types by non-parametric Kruskal-Wallis test (P<0.05).

*COL* and *ITG* mRNA expression (median)	High-grade neuroendocrine tumors		Carcinoid tumors		ControlLung normal tissue	P-value
	SCLC	LCNEC	AC	TC		
***COL1A2***	15.47	2.60	0.65	0.65	1.77	0.004
***COL3A1***	9.46	5.05	0.59	1.18	2.97	0.004
***COL5A2***	15.54	3.24	0.64	0.65	2.58	0.003
***ITGA5***	4.63	3.49	0.10	0.78	1.54	0.009
***ITGAV***	5.48	3.67	0.92	1.85	1.82	0.028
***ITGB1***	7.28	1.37	0.46	1.84	1.83	0.057

PNENs, pulmonary neuroendocrine neoplasms; SCLC, small cell lung carcinoma; LCNEC, large cell neuroendocrine carcinoma; AC, atypical carcinoid; TC, typical carcinoid; COL, collagen; ITG, integrin.

Genes from the COL family seemed to play a particularly important role in several metrics. First, a significant association was found between *COL* genes (*COL1A2*, *COL3A1*, and *COL5A2*) and *ITGA5*, *ITGAV*, and *ITGB1* (P<0.05) (**Table 3** **)**, but a correlation also emerged between *COL* and *ITG* expressions and disease progression profile. For instance, the overexpression of *COL1A2*, *COL3A1*,and *COL5A2* in the TME was strongly associated with T stage, N stage, and pathological stage (P<0.05, in all three cases). M stage, in turn, was only significantly associated with *COL3A1* (P=0.04). Similarly, the overexpression of *ITGA5* and *ITGAV* were strongly associated with N stage, and pathological stage, (P<0.05), while *ITGB1* was associated with T stage and N stage, (P=0.03 and P=0.00, respectively), as shown in **Table 4** and **Supplementary Figures 1** and **2**.

**Table 3:** Correlation between *COL* and *ITG* gene expression in TME by non-parametric Kendall tau-b correlation coefficient (P<0.05).

Variables	ρ	*P*-value
***COL1A2***		
* ITGA5*	0.645	<0.001
* ITGAV*	0.522	<0.001
* ITGB1*	0.616	<0.001
***COL3A1***		
* ITGA5*	0.471	0.001
* ITGAV*	0.406	0.005
* ITGB1*	0.514	<0.001
***COL5A2***		
* ITGA5*	0.536	<0.001
* ITGAV*	0.500	0.001
* ITGB1*	0.594	<0.001

COL, collagen; ITG, integrin.

**Table 4:** Statistical analysis by non-parametric Spearman’s rank correlation coefficient (P<0.05) between *COL* and *ITG* gene expression and the disease progression profile.

Variables	ρ	p-Value
***COL1A2***		
T Stage†	0.58	0.017
N Stage†	0.75	0.001
M Stage†	0.39	0.091
Early (I/II) *vs.* Advanced stage (III/IV)	0.69	0.000
***COL3A1***		
T Stage†	0.66	0.005
N Stage†	0.67	0.004
M Stage†	0.45	0.049
Early (I/II) *vs.* Advanced stage (III/IV)	0.68	0.000
***COL5A2***		
T Stage†	0.60	0.012
N Stage†	0.82	0.000
M Stage†	0.41	0.075
Early (I/II) *vs.* Advanced stage (III/IV)	0.72	0.000
***ITGA5***		
T Stage†	0.33	0.209
N Stage†	0.71	0.002
M Stage†	0.28	0.284
Early (I/II) *vs.* Advanced stage (III/IV)	0.65	0.000
***ITGAV***		
T Stage†	0.29	0.261
N Stage†	0.63	0.008
M Stage†	0.23	0.324
Early (I/II) *vs.* Advanced stage (III/IV)	0.53	0.007
***ITGB1***		
T Stage†	0.52	0.036
N Stage†	0.75	0.001
M Stage†	0.31	0.184
Early (I/II) *vs.* Advanced stage (III/IV)	0.34	0.102

^†^8th International Association for the Study of Lung Cancer (14).

Functionally, the six fibrogenic genes (*COL1A2*, *COL3A1*, *COL5A2*, *ITGA5*, *ITGAV* and *ITGB1*) were involved in biological pathways associated with transmission of molecular signals to drive the activation of the resident fibroblasts into cancer-associated fibroblasts (CAFs) able to secrete and assembly the collagen fibers (cross-linking), and promote a desmoplastic reaction characterized by increased stiffness of the stroma for invasion and metastasis. In the Metascape analysis, the GO produced a list of top-level biological process and a heatmap of enriched terms related to the input genes, which included: “PID integrin 1 pathway”, “extracellular matrix organization”, “assembly of collagen fibrils and other multimeric structures”, and “ECM-receptor interaction” (**Supplementary Figure 3A**). We then consulted the enrichment analysis in DisGeNET, where these six matricellular-associated genes seem to be involved in several diseases, as shown in **Supplementary Figure 3B**. Finally, **Supplementary Figure 3C** shows the PPI network formed in relation to biological processes; these enriched terms were produced using the following databases: BioGrid6, InWeb_IM7, OmniPath.

The PPI network with the proteins that are encoded by these genes using the STRING database included the 11 functional partners with the highest interaction score, namely COL1A1, COL1A2, COL3A1, COL5A2, LUM, ITGA5, ITGAV, ITGB1, ITGB3, ITGB6 and ITGB8. The edges represent protein-protein associations that contribute to a shared function and involve only those proteins with a high edge score (confidence ≥ 0.9). These proteins’ molecular organization can be visualized as a network of differentially connected nodes shown in **Supplementary Figure 4**.

### Validation Cohort: Independent Validation of Differentially Expressed *COL* and *ITG* Genes

The expression of *COL1A2*, *COL3A1*, *COL5A2*, *ITGA5*, and *ITGB1* in SCLC, LCNEC, and typical carcinoid samples in the GSE1037 profile, as compared with normal samples, were determined using a GEO2R online analyzer (log FC>2 and adjusted P<0.05) and then compared to the data from our cohort. We observed a similar expression of collagens between the groups, mainly in the PNEC group, although the gene expression in our cohort was more expressive. However, some differences were detected in the expression of integrins. The expression of *ITGA5* was inversely proportional between the groups: according to our data, this gene was overexpressed in PNECs and underexpressed in PNETs, whereas the GSE1037 data showed the opposite pattern. Similarly, while in our data *ITGB1* was overexpressed mainly in PNECs, in GSE1037 it was mostly overexpressed in PNETs. A heatmap between fold changes was then established to show genes whose expression differed between our gene profile data (**Supplementary Figure 5A**) and the GSE1037 gene profile data (**Supplementary Figure 5B**).

### *COL* and *ITG* Modulate Overall Survival and Risk of Death

A preliminary examination of the Kaplan-Meier survival curves generated in this study demonstrated that patients with advanced stage of disease (III/IV) had worse overall survival (OS) compared to those in early stage (I/II), 12 *vs.* 151 months (P=0.000). Thus, we coded the overall pathological stage as a single dummy variable with a value of 1 for stages I and II, and a value of 2 for stages III and IV. The results of the Cox model analysis are found in **Table 5**. Univariate Cox proportional hazards analyses showed that tobacco history, clinical stage, distant metastasis, and the expression of collagen and integrin – with exception to *ITGAV* – were significant predictors for OS and risk of death. These variables were accounted for in the multivariate analysis, in order to explore their independent predictive effect for OS.

**Table 5:** Variables associated with overall survival of patients diagnosed with PNENs.

	Univariate Analysis^a^			Multivariate Analysis^b^	
	HR^c^ (95% CI)	HR^c^	P-value	HR^c^ (95% CI)^d^	P*-*value
**Clinicopathological Characteristics**					
Age, median (yrs): <56 vs ≥56	0.55 (0.18-1.65)	-0.59	0.291		
Gender					
* Male*	0.88 (0.30-2.56)	-0.12	0.825		
Tobacco History					
* Yes*	5.19 (1.15-23.31)	1.64	0.032	6.20 (0.68-56.63)	0.106
Pathological Stage (Early I/II vs Advanced III/IV)	0.05 (0.00-0.41)	-2.96	0.005		
Early (I/II) †					
Lymph Node Metastasis †					
* Absense*	0.00 (0.00-1.42)	-5.09	0.067		
Distant Metastasis (M) †					
* Absence*	0.21 (0.06-0.74)	-1.54	0.016	0.19 (0.04-0.88)	0.034
Chemotherapy	0.64 (0.19-2.07)				
* Yes*		-0.44	0.457		
Radiotherapy	1.27 (0.39-4.10)	0.243			
* Yes*			0.684		
**Gene expression of *COL* and *ITG* (median)**					
***COL1A2* mRNA**					
<2.59	0.14 (0.04-0.55)	-1.90	0.004		
≥2.59 (*reference*)					
***COL3A1* mRNA**					
<2.95	0.15 (0.04-0.58)	-1.83	0.006		
≥2.95 (*reference*)					
***COL5A2* mRNA**					
<5.15	0.27 (0.08-0.87)	-1.29	0.029	0.28 (0.05-1.63)	0.160
≥5.15 (*reference*)					
***ITGA5* mRNA**					
<3.06	0.19 (0.05-0.69)	-1.66	0.012	0.24 (0.04-1.40)	0.115
≥3.06 (*reference*)					
***ITGAV* mRNA**					
<3.65	0.33 (0.10-1.11)	-1.08	0.076		
≥3.65 (*reference*)					
***ITGB1* mRNA**					
<3.64	0.18 (0.05-0.70)	-1.67	0.013	0.07 (0.91-54.42)	0.060
≥3.64 (*reference*)					

^a^Univariate analysis was carried out without any adjustment in order to generate hazard ratios with confidence intervals for individual risk for each of the parameters on survival; ^b^Multivariate analysis was carried out to analyze the effects of several risk parameters on survival; ^c^HR, hazard ratio (β coefficient); ^d^CI, confidence interval. Univariate and multivariate analysis employed a Cox proportional hazards model. Chi-square 16.38, P=0.006.

^†^8th International Association for the Study of Lung Cancer (14).

**Figure 2:**
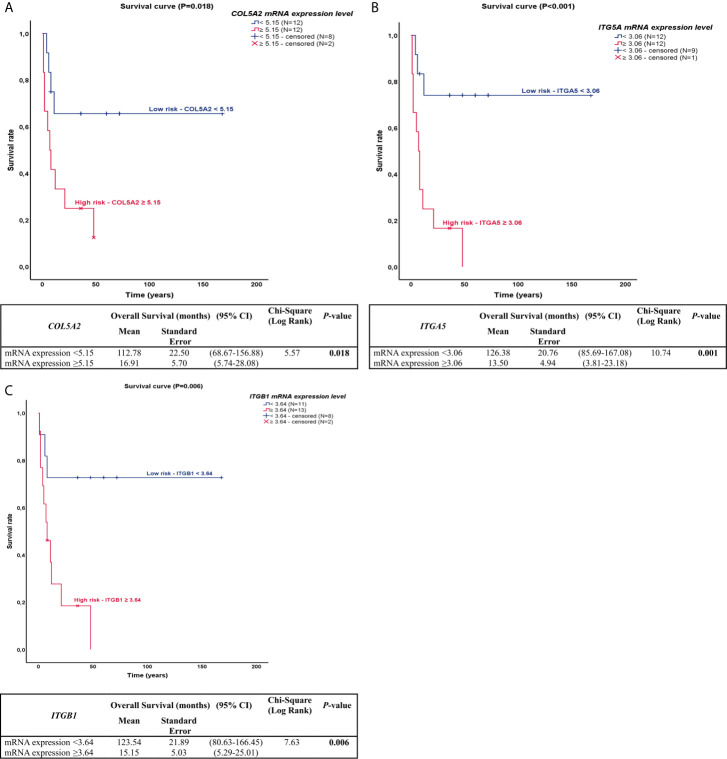
Kaplan-Meier plots for overall survival for PNEN patients according to the difference in *COL* and *ITG* gene expressions. **(A)** In the Kaplan-Meier survival curve of *COL5A2*, patients who had high expression (top of the curve) presented lower survival rate than those who had lower expression (bottom of the curve), 112.78 *vs* 16.61 months, (P=0.018); **(B)** In the Kaplan-Meier survival curve of *ITGA5*, patients who had high expression (top of the curve) presented lower survival rate than those who had lower expression (bottom of the curve), 126.38 *vs* 13.50 months, (P=0.001); **(C)** In the Kaplan-Meier survival curve of *ITGB1*, patients who had high expression (top of the curve) presented lower survival rate than those who had lower expression (bottom of the curve), 123.54 *vs* 15.15 months, (P=0.006).

The most effective survival model, under the conditions of the study, was controlled by tobacco history, distant metastasis, and *COL5A2*, *ITGA5*, and *ITGB1* expression, all of them variables represented as co-dependent factors in the model. Distant metastasis was also presented as an independent factor of OS and risk of death (P=0.034). The chi-square including the covariates was 16.38 (P<0.01). In the Kaplan-Meier plots, the top curves represent the group with low expression of *COL5A2*, *ITGA5*, and *ITGB1*, whose median survival was quite long (112.78, 126.38, and 113.54 months, respectively). By contrast, patients with high expression of *COL5A2*, *ITGA5*, and *ITGB1* (bottom curves) had a median survival time of just 16.91, 13.50, and 15.15 months, respectively (P<0.05, by log-rank test), as shown in **Figure 2**.

## Discussion

In the present study, we evaluated gene expression profiles using a set of twenty-four patients with surgically resected PNENs, including SCLC, and identified six fibrogenic genes: *COL1A2, COL3A1, COL5A2, ITGA5, ITGAV*, and *ITGB1* up-regulated in PNECs and down-regulated in PNETs. We used two approaches where we first examined the six candidate fibrogenic genes using a whole-genome screen, and subsequently, validated the upregulation of these six genes in a similar independent validation cohort using *in silico* analysis. We additionally observed that low expression of three of these genes (*COL5A2*, *ITGA5*, and *ITGB1*) were significantly associated with metastatic-free and overall survival in PNENs. Our findings suggest that incorporation of collagen/integrin gene expression profile to routine genome-wide examination of biomarkers helps to predict metastasis in pulmonary neuroendocrine neoplasms and may be a promising tool to select and customize therapy.

Although treatment options for metastatic PNENs have increased over the past decade, mortality and 5-year survival remain little altered for PNETs (19) and PNECs (20, 21). Molecular studies identified somatic mutations, somatic copy numbers and pathway alterations in primary PNENs tumors (6–8); however, less is known regarding the effects of fibrogenic genes over the more lethal and therapeutically relevant stroma-associated fibrosis. Thus, studies that interrogate fibrogenic genes in tumors are critical to understanding the biology of invasion and metastasis in these tumors, the major cause of patient mortality.

The process of cancer cell invasion and metastasis undoubtedly comprises a series of complex, sequential stages, but among these the high collagen and integrins expression levels signalized by cancer-associated fibroblasts (CAFs) resulting in stroma-associated fibrosis is thought to be important because facilitates the migration of tumor cells and penetration of tumor by blood vessels (22–26). In order to understand the roles of fibrotic reaction in metastatic process, we explored the mRNA level of the different fibrillar collagens. We found that *COL1A2*, *COL3A1*, *COL5A2*, tumor mediators targeting of relevant structural components of the extracellular matrix (ECM) were activated to drive CAFs to synthetize fibrillar collagen creating an aberrant microenvironment (22). Convincing reports propose that a normal microenvironment avoids premalignant cells from developing into cancer, whereas an atypical or scarring repair–associated microenvironment can be tumor-promoting (27). The disruption in tissue homeostasis activates matrix fibroblasts into CAFs to synthetize collagen I, III and V (22), which in turn lead to a fibrotic repair of tumor stroma which is a major player in the development and progression of many cancers, including lung cancer, pancreas, breast, and hepatic carcinomas (28–30).

In the above scenario, fibrillar collagen types are the key actors in tumor stroma-associated fibrosis (also called desmoplasia), which is defined as a fibrotic state characterized by an excessive synthesis, deposition and remodeling of fibrillar collagens surrounding the tumor (31, 32). Collagen represents the most abundant ECM protein and collagen I, III and V deposition have been associated with increased desmoplasia leading to increased incidence of tumor formation and metastasis (33). A study found a gene expression signature that distinguished primary and metastatic adenocarcinomas and predicts the metastatic probability of these tumors; and a considerable proportion of the gene-expression signature described was composed of tumor *COL* gene expression, such as *COL1A1* and *COL1A2*, both drivers of CAFs to synthesize collagen I fibers deposition resulting in stroma-associated fibrosis (34).

We show that, additionally to *COL1A2, COL3A1* and *COL5A2* were also up regulated in PNECs and downregulated in PNETs. Although collagen V is a minor constituent of the ECM compared to collagen I and III, collagen V is essential for fibrillogenesis, as its deletion leads to inability of collagen fibril assembly resulting in fibrotic stroma (35). Moreover, other studies observed that increased expression of collagen V individualizing malignant cells conferred an abnormal tumor stroma-associated fibrosis to local invasion and recurrence in malignant mesothelioma (36, 37). In lung adenocarcinoma and breast cancer was observed that decreased expression of collagen V organized in an irregular texture of thin fibers involving large groups of malignant cells, facilitated invasion in a poor tumor stroma-associated fibrosis (36), also coinciding with previous work by Souza et al. (38). Hitherto, it was demonstrated that increased tumor stroma-associated fibrosis proportion in lung adenocarcinoma predicted a low risk of metastasis (39).

According to the literature, this double edge sword of fibrosis in cancer due to collagen deposition is that this protein evokes multiple, and sometimes opposite, cellular responses, depending on the cell type (40). For instance, while collagen I and collagen V have been proven to represent an optimal substrate in fibrotic stroma for the attachment and growth of certain tumor cell types (41), this collagen species plays an antiadhesive and antiproliferative role in breast cancer cells (42). Furthermore, we showed a bimodal behavior of *COL1A2, COL3A1* and *COL5A2* up-regulated in PNECs and down-regulated in PNETs. As PNENs are tumors with a limited stroma, the question is how the tumor cells migrate to gain access into vessels? We inferred that fibrotic genes in PNENs drives the migration of cells depending on collagen density in tumor stroma. In PNETs, down-regulated fibrotic genes drive low collagen synthesis by CAFs, resulting in a loose fibrosis allowing tumor cells moving fast using pseudopodial protrusions as previously reported (43). In contrast, upregulation of fibrotic genes in PNECs increases deposition of fibrillar collagens making cell movements rely more extensively after collagenase cleavage of collagen fibers (44).

We have also found a strong quantitative relationship between *COL1A2, COL3A1, COL5A2* and *ITGA5, ITGAV* and *ITGB1* genes. As expected functionally, *COL1A2, COL3A1, COL5A2, ITGA5, ITGAV*, and *ITGB1* genes were mostly involved in ECM remodeling, collagen fibers deposition, fibril harmonization, and cell adhesion. Elevated *ITG* signaling activates *COL* genes to deposit collagen proteins (fibers) in ECM creating a fibrotic stroma. Protein-protein interactions network showed 11 functional partners with the highest interaction score, namely COL1A1, COL1A2, COL3A1, COL5A2, LUM, ITGA5, ITGAV, ITGB1, ITGB3, ITGB6 and ITGB8. This signaling facilitates movement and migration of tumor cells between fibrotic stroma (45). Furthermore, increased stroma stiffness, a consequence of elevated expression of *COL* genes and collagen fibers deposition, causes activation of MAPKs and Rho-GTPases also *via ITG* signaling. These pathways are strong stimulators of fibrotic reaction for tumor migration, invasion, and metastasis (46). Integrin *ITGB1* promotes cell invasion by sensitizing cancer cells to the changes in the fibrotic stroma (47), while Rho-GTPases are indispensable in the regulation of cell migration and control of multiple aspects of M phase and G1 progression of the cell cycle (46).

Recent studies have shown that several *COL* and *ITG* genes are increased in a variety of tumors, and have been associated with unfavorable outcomes. Among these, *COL1A2*, *COL3A1*, *COL5A2*, *ITGA5*, *ITGAV*, and *ITGB1* have been especially noted (48–53). Therefore, for all these reasons, we realize that *COL1A2*, *COL3A1*, *COL5A2*, *ITGA5*, *ITGAV*, and *ITGB1* genes expression provide important predictive information about metastatic-free and overall survival in PNENs and our results now confirm the predictive importance of *COL* and *ITG* genes in PNENs. Whereas prior studies about lung cancer were able to show a significant relationship between *COL5A2*, *ITGA5*, *ITGAV*, and *ITGB1* gene expression only in non-small cell lung cancer (54–58), our results suggest that *COL5A2*, *ITGA5*, and *ITGB1* expression in PNENs, used as co-dependent variables, provide more information about the risk of metastasis and overall survival than does pathological stage. Moreover, the predictive value of *COL5A2, ITGA5*, and *ITGB1* expression in PNENs persisted in the subset of patients with pathological stage I and II. In this context, we were able to identify two groups: patients with an expected low risk of metastasis and better overall survival *versus* patients with an expected high risk of metastasis and poor overall survival. Therefore, evaluation of the primary tumor for *COL* and *ITG* give us tools to guide the use of targeted therapy in patients expected to fail after surgical resection of PNENs.

In summary, the results presented herein provide important molecular evidence that collagen/integrin profiling are involved in the stroma-associated fibrosis to facilitate metastatic potential of PNENs. Specifically, our study indicates that collagen/integrin genes are up-regulated in PNECs and down- regulated in PNETs and thus potentially offer insight into novel therapeutic targets. Overall, these fibrogenic genes may represent partially an ECM ‘remodeling’ program to drive metastatic establishment. Preclinical studies are warranted, therapeutically, to select target up-regulated fibrogenic genes, mainly in SCLC, while maintaining proper ECM integrity in normal tissue.

## Data Availability Statement

The original contributions presented in the study are publicly available. This data can be found here: https://www.ncbi.nlm.nih.gov/geo/query/acc.cgi?acc=GSE181381.

## Ethics Statement

The studies involving human participants were reviewed and approved by Ethics Committee for Research Project Analysis (CAPPesq) of the Hospital das Clínicas, Faculty of Medicine of University of São Paulo. Written informed consent for participation was not required for this study in accordance with the national legislation and the institutional requirements.

## Author Contributions

Conception and design: VC and TP. Writing, review, and editing: VC, TP, CB, and JM-R. Data analysis and interpretation: VC, TP, CB, JM-R, AV, and WT. Statistical analysis: VC and TP. Provision of study materials or patients: AA’b. Administrative support: VC. All authors contributed to the article and approved the submitted version.

## Funding

This work was supported by Sao Paulo Research Foundation (FAPESP; 2018/20403-6, 2019/12151-0), the National Council for Scientific and Technological Development (CNPq; 483005/2012-6), and Coordenação de Aperfeiçoamento de Pessoal de Nível Superior - Brasil (CAPES; Finance Code 001).

## Conflict of Interest

The authors declare that the research was conducted in the absence of any commercial or financial relationships that could be construed as a potential conflict of interest.

## Publisher’s Note

All claims expressed in this article are solely those of the authors and do not necessarily represent those of their affiliated organizations, or those of the publisher, the editors and the reviewers. Any product that may be evaluated in this article, or claim that may be made by its manufacturer, is not guaranteed or endorsed by the publisher.

## Footnotes

1 http://www.ncbi.nlm.nih.gov/geo/

2 http://www.Heatmapper.ca/expression/

## Supplementary Material

Supplementary Figure 1 Box plot of *COL* gene expression among tumor staging (TNM) and Pathological stage – Early (I/II) *vs* Advanced (III/IV) in a log scale. The top and bottom of the box plot represents the 25th and 75th percentile range. The line across the box shows the median of gene expression and the top and bottom bars show the maximum and minimum values, outliers were showed. The association between *COL* gene expression and tumor staging (TNM) and Pathological stage was calculated by non-parametric Spearman’s rank correlation coefficient. **(A)** *COL1A2* mRNA expression level among T stage; (P=0.017); **(C)** *COL1A2* mRNA expression level among N stage (P=0.001); **(C)** *COL1A2* mRNA expression level among M stage (P=0.091); **(D)** *COL1A2* mRNA expression level among Pathological stage (P=0.000); **(E)** *COL3A1* mRNA expression level among T stage (P=0.005); **(F)** *COL3A1* mRNA expression level among N stage (P=0.004); **(G)** *COL3A1* mRNA expression level among M stage (P=0.049); **(H)** *COL3A1* mRNA expression level among Pathological stage (P=0.000); **(I)** *COL5A2* mRNA expression level among T stage (P=0.012); **(J)** *COL5A2* mRNA expression level among N stage (P=0.000); **(K)** *COL5A2* mRNA expression level among M stage (P=0.075); **(L)** *COL5A2* mRNA expression level among Pathological stage (P=0.000).

Supplementary Figure 2 Box plot of *ITG* gene expression among tumor staging (TNM) and Pathological stage – Early (I/II) *vs* Advanced (III/IV) in a log scale. The top and bottom of the box plot represents the 25th and 75th percentile range. The line across the box shows the median of gene expression and the top and bottom bars show the maximum and minimum values, outliers were showed. The association between *ITG* gene expression and tumor staging (TNM) and Pathological stage was calculated by non-parametric Spearman’s rank correlation coefficient. **(A)** ITGA5 mRNA expression level among T stage (P=0.209); **(B)** *ITGA5* mRNA expression level among N stage (0.002); **(C)** *ITGA5* mRNA expression level among M stage (P=0.284); **(D)** *ITGA5* mRNA expression level among Pathological stage (P=0.000); **(E)** *ITGAV* mRNA expression level among T stage (P=0.261); **(F)** *ITGAV* mRNA expression level among N stage (P=0.008); **(G)** *ITGAV* mRNA expression level among M stage (P=0.324); **(H)** *ITGAV* mRNA expression level among Pathological stage (P=0.007); **(I)** *ITGB1* mRNA expression level among T stage (P=0.036); **(J)** *ITGB1* mRNA expression level among N stage (P=0.001); **(K)** *ITGB1* mRNA expression level among M stage (P=0.184); **(L)** *ITGB1* mRNA expression level among Pathological stage (P=0.102).

Supplementary Figure 3 Enrichment analysis of 6 ECM-related genes using Metascape. **(A)** Heatmap of enriched terms across input gene lists, colored by P-values. **(B)** Summary of enrichment analysis in DisGeNET colored by P-values. **(C)** Protein-protein interaction network.

Supplementary Figure 4 Cluster analysis of the PPI network using the STRING database for ECM protein interactions. The network included the 11 functional partners with the highest interaction confidence score, namely, COL1A1, COL1A2, COL3A1, COL5A2, LUM, ITGA5, ITGAV, ITGB1, ITGB3, ITGB6 and ITGB8, (score ≥ 0.9).

Supplementary Figure 5 Validation cohort: Heatmap for comparison between differentially expressed *COL* and *ITG* genes across PNENs histological subtypes in the GSE1037 profile public data and in our data. **(A)** Heatmap of *COL* and *ITG* genes differentially expressed among PNENs histological subtypes in our cohort of 24 patients. **(B)** Heatmap of *COL* and *ITG* genes differentially expressed among PNENs histological subtypes in the GSE1037 public profile data of 35 patients.
