# Supplementary figures and images for: The Fibrosis-Targeted Collagen/Integrins Gene Profile Predicts Risk of Metastasis in Pulmonary Neuroendocrine Neoplasms
Source: Front Oncol. 2021 Aug 11;11:706141. doi: 10.3389/fonc.2021.706141 (PMC8385766; doi:10.3389/fonc.2021.706141)

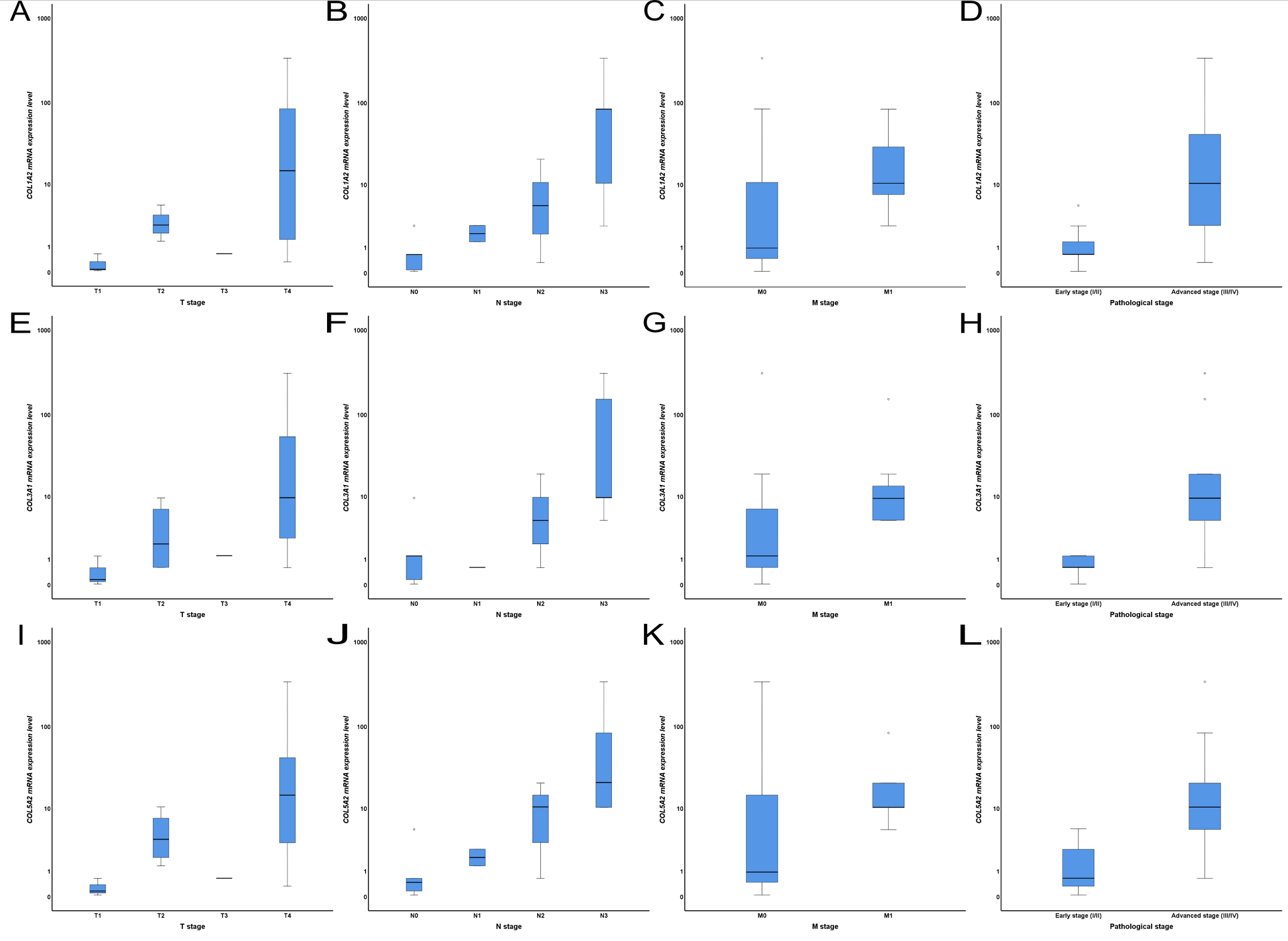

Supplement: Supplementary Figure 1 — Box plot of COL gene expression among tumor staging (TNM) and Pathological stage – Early (I/II) vs Advanced (III/IV) in a log scale. The top and bottom of the box plot represents the 25th and 75th percentile range. The line across the box shows the median of gene expression and the top and bottom bars show the maximum and minimum values, outliers were showed. The association between COL gene expression and tumor staging (TNM) and Pathological stage was calculated by non-parametric Spearman’s rank correlation coefficient. (A) COL1A2 mRNA expression level among T stage; (P=0.017); (C) COL1A2 mRNA expression level among N stage (P=0.001); (C) COL1A2 mRNA expression level among M stage (P=0.091); (D) COL1A2 mRNA expression level among Pathological stage (P=0.000); (E) COL3A1 mRNA expression level among T stage (P=0.005); (F) COL3A1 mRNA expression level among N stage (P=0.004); (G) COL3A1 mRNA expression level among M stage (P=0.049); (H) COL3A1 mRNA expression level among Pathological stage (P=0.000); (I) COL5A2 mRNA expression level among T stage (P=0.012); (J) COL5A2 mRNA expression level among N stage (P=0.000); (K) COL5A2 mRNA expression level among M stage (P=0.075); (L) COL5A2 mRNA expression level among Pathological stage (P=0.000). [file Image_1.tiff]

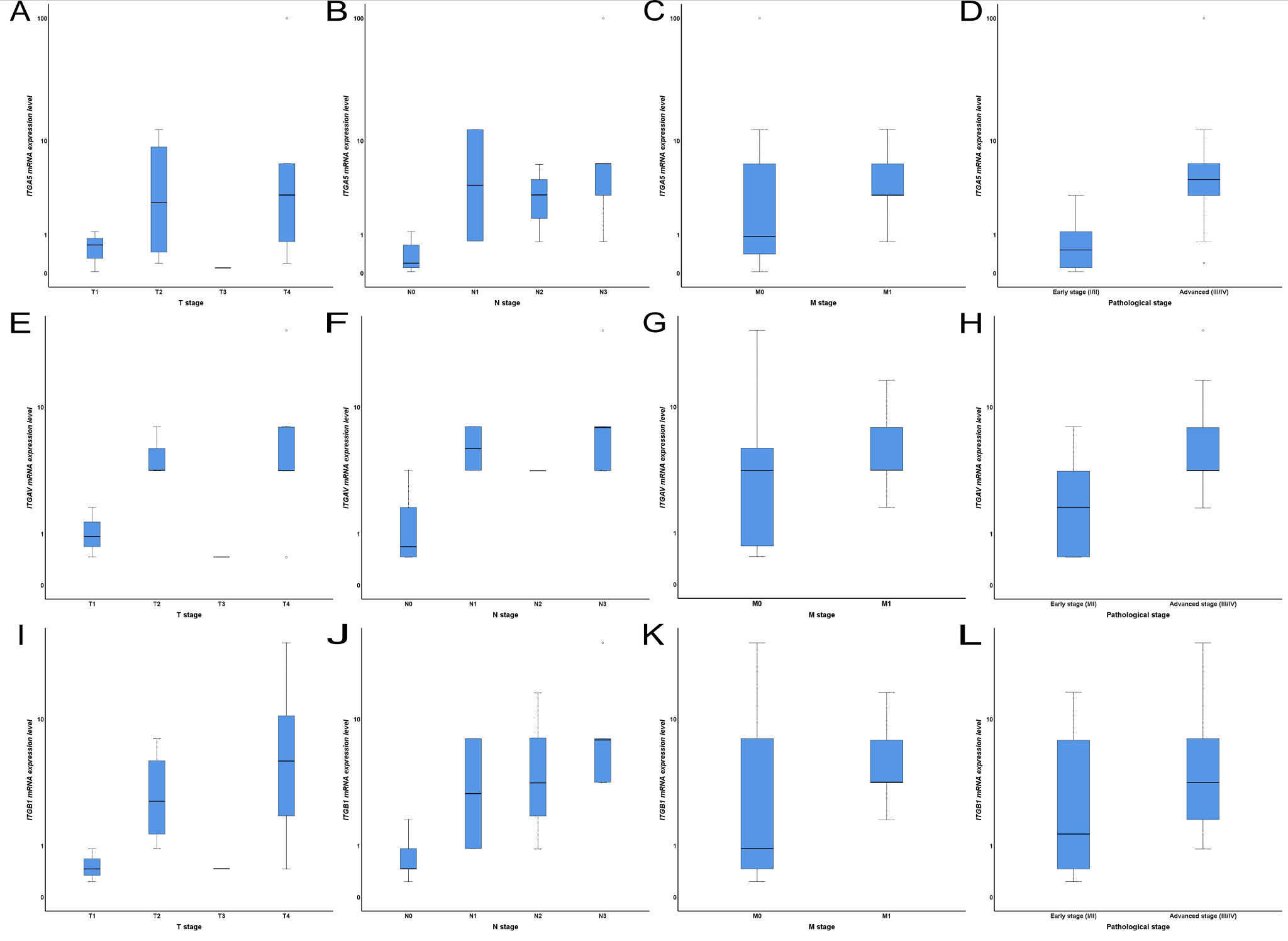

Supplement: Supplementary Figure 2 — Box plot of ITG gene expression among tumor staging (TNM) and Pathological stage – Early (I/II) vs Advanced (III/IV) in a log scale. The top and bottom of the box plot represents the 25th and 75th percentile range. The line across the box shows the median of gene expression and the top and bottom bars show the maximum and minimum values, outliers were showed. The association between ITG gene expression and tumor staging (TNM) and Pathological stage was calculated by non-parametric Spearman’s rank correlation coefficient. (A) ITGA5 mRNA expression level among T stage (P=0.209); (B) ITGA5 mRNA expression level among N stage (0.002); (C) ITGA5 mRNA expression level among M stage (P=0.284); (D) ITGA5 mRNA expression level among Pathological stage (P=0.000); (E) ITGAV mRNA expression level among T stage (P=0.261); (F) ITGAV mRNA expression level among N stage (P=0.008); (G) ITGAV mRNA expression level among M stage (P=0.324); (H) ITGAV mRNA expression level among Pathological stage (P=0.007); (I) ITGB1 mRNA expression level among T stage (P=0.036); (J) ITGB1 mRNA expression level among N stage (P=0.001); (K) ITGB1 mRNA expression level among M stage (P=0.184); (L) ITGB1 mRNA expression level among Pathological stage (P=0.102). [file Image_2.tiff]

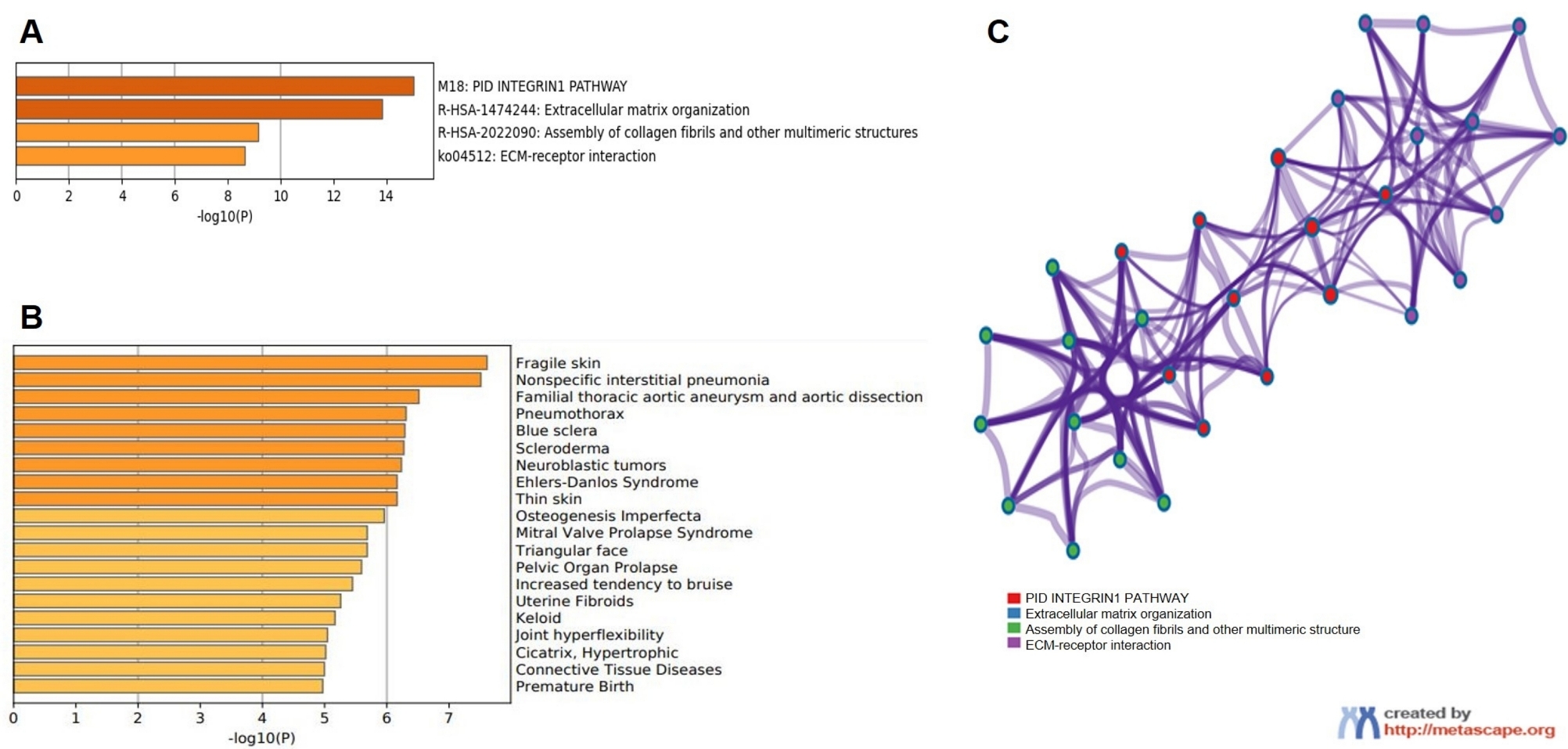

Supplement: Supplementary Figure 3 — Enrichment analysis of 6 ECM-related genes using Metascape. (A) Heatmap of enriched terms across input gene lists, colored by P-values. (B) Summary of enrichment analysis in DisGeNET colored by P-values. (C) Protein-protein interaction network. [file Image_3.jpeg]

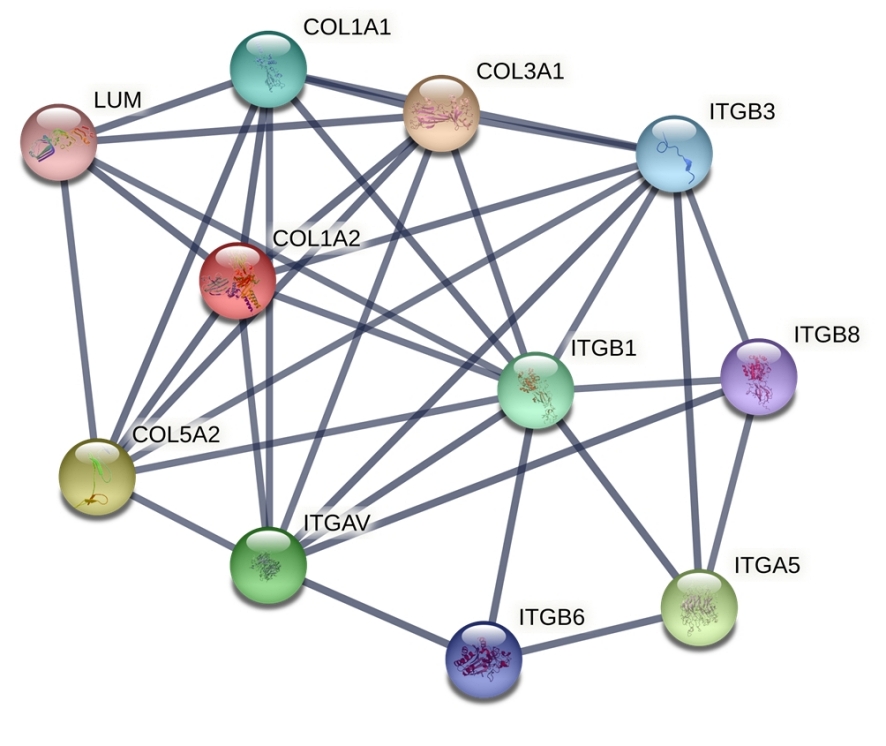

Supplement: Supplementary Figure 4 — Cluster analysis of the PPI network using the STRING database for ECM protein interactions. The network included the 11 functional partners with the highest interaction confidence score, namely, COL1A1, COL1A2, COL3A1, COL5A2, LUM, ITGA5, ITGAV, ITGB1, ITGB3, ITGB6 and ITGB8, (score ≥ 0.9). [file Image_4.jpeg]

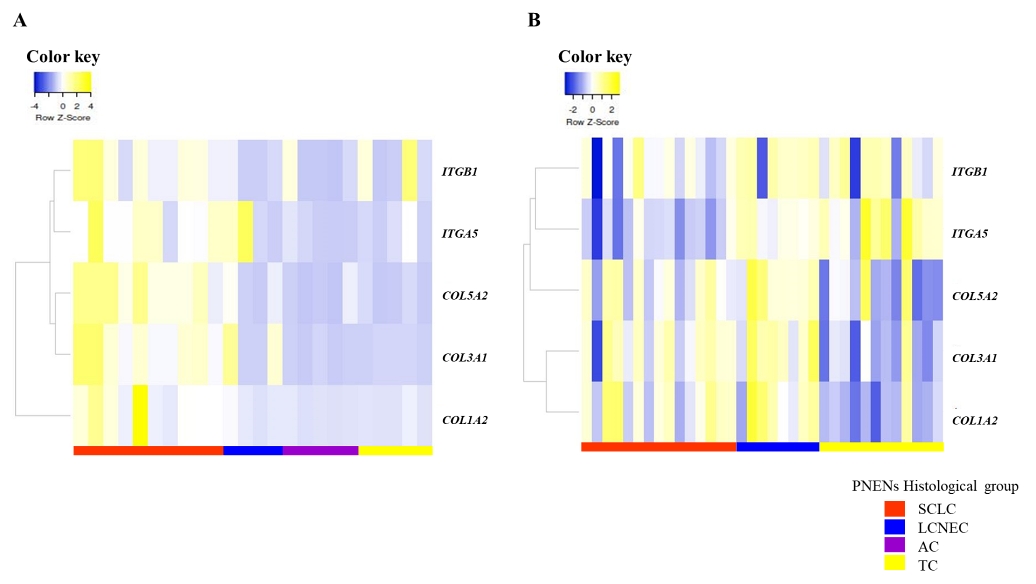

Supplement: Supplementary Figure 5 — Validation cohort: Heatmap for comparison between differentially expressed COL and ITG genes across PNENs histological subtypes in the GSE1037 profile public data and in our data. (A) Heatmap of COL and ITG genes differentially expressed among PNENs histological subtypes in our cohort of 24 patients. (B) Heatmap of COL and ITG genes differentially expressed among PNENs histological subtypes in the GSE1037 public profile data of 35 patients. [file Image_5.jpeg]
